# Supplementary material for: Ethnic Variations in the Levels of Bone Biomarkers (Osteoprostegerin, Receptor Activator of Nuclear Factor Kappa-Β Ligand and Glycoprotein Non-Metastatic Melanoma Protein B) in People with Type 2 Diabetes
Source: Biomedicines. 2024 May 6;12(5):1019. doi: 10.3390/biomedicines12051019 (PMC11117910; doi:10.3390/biomedicines12051019)
Supplement: Supplementary file 1 [file biomedicines-12-01019-s001.zip › biomedicines-2935944-supplementary.pdf]

Table S1. Demographic distribution of the population stratified by ethnicity.

ARABS		%	ASIANS		%
EGYPTIAN	77	8.9	AFGHANIS	8	0.8
IRANIAN	18	2.1	BANGLADE	28	2.7
IRAQI	7	0.8	INDIAN	504	48.9
JORDANIA	51	5.9	INDONESI	3	0.3
KUWAITI	569	65.4	JAPANIES	2	0.2
LEBANESE	47	5.4	NEPAL	4	0.4
MORROCO	1	0.1	PAKISTAN	90	8.7
PALASTIN	5	0.6	PHILIPIN	351	34.1
SAUDI	2	0.2	SRILANKA	26	2.5
SUDANIAN	1	0.1	VEITNAM	6	0.6
SYRIAN	74	8.5	OTHERS	8	0.8
YEMAN	4	0.5	TOTAL	1030	100.0
OTHERS	14	1.6			
TOTAL	870	100.0			
MISSING	29				

Reported as number of individuals of certain citizenship, and percentage within the ethnic category.
